# Thermal transport in nanocrystalline Si and SiGe by *ab initio* based Monte Carlo simulation

**DOI:** 10.1038/srep44254

**Published:** 2017-03-14

**Authors:** Lina Yang, Austin J. Minnich

**Affiliations:** 1 Division of Engineering and Applied Science, California Institute of Technology, Pasadena, California 91125, United States

## Abstract

Nanocrystalline thermoelectric materials based on Si have long been of interest because Si is earth-abundant, inexpensive, and non-toxic. However, a poor understanding of phonon grain boundary scattering and its effect on thermal conductivity has impeded efforts to improve the thermoelectric figure of merit. Here, we report an ab-initio based computational study of thermal transport in nanocrystalline Si-based materials using a variance-reduced Monte Carlo method with the full phonon dispersion and intrinsic lifetimes from first-principles as input. By fitting the transmission profile of grain boundaries, we obtain excellent agreement with experimental thermal conductivity of nanocrystalline Si [Wang *et al*. Nano Letters 11, 2206 (2011)]. Based on these calculations, we examine phonon transport in nanocrystalline SiGe alloys with ab-initio electron-phonon scattering rates. Our calculations show that low energy phonons still transport substantial amounts of heat in these materials, despite scattering by electron-phonon interactions, due to the high transmission of phonons at grain boundaries, and thus improvements in *ZT* are still possible by disrupting these modes. This work demonstrates the important insights into phonon transport that can be obtained using ab-initio based Monte Carlo simulations in complex nanostructured materials.

Thermoelectric (TE) materials are of interest for energy applications such as waste heat recovery and quiet, reliable refrigeration^1^,^2^,^3^,^4^. The performance of TE devices is determined by the figure of merit 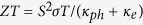, where *S* is the Seebeck coefficient, *σ* is the electrical conductivity, *T* is the absolute temperature, and *κ*_*e*_ and *κ*_*ph*_ are the electronic and lattice thermal conductivity^5^. Substantial increases in *ZT* have been achieved in recent years by reducing lattice thermal conductivity in nanostructured materials while minimizing decreases in electronic properties^4^,^6^.

Nanocrystalline materials, or polycrystals with nanoscale grain size, in particular have been demonstrated as efficient TE materials in bulk form^4^,^6^,^7^,^8^,^9^,^10^,^11^,^12^. The thermoelectric efficiency is improved by the reduction of lattice thermal conductivity due to phonon grain boundary scattering when the phonon mean free paths are comparable to the grain size. Of the many materials that have been processed into nanocrystalline form, nanocrystalline Si is a promising candidate as it is inexpensive and not toxic^7^. However, the peak *ZT* achieved in nanostructured silicon is around 0.7, still lower than those of champion thermoelectric materials primarily because of its high thermal conductivity.

Further improving *ZT* requires a detailed understanding of phonon grain boundary scattering and its effect relative to other scattering mechanisms such as phonon-phonon and electron-phonon scattering. Both computation and experiment have been used extensively to investigate phonon transmission across grain boundaries. Many studies using atomic calculations reported that the transmission of phonons across the grain boundaries depends on the phonon frequency and sharply decreases as the frequency increases^13^,^14^,^15^,^16^. Experimental work by Wang *et al*. reported a *T*^2^ temperature dependent thermal conductivity of nanocrystalline silicon, which further confirms this trend^17^. Recently, the transmission coefficients between Si and Al were reported by Hua *et al*. using measurements from the time-domain thermoreflectance method and ab-initio phonon transport modeling^18^. They found that the transmission coefficients were close to unity for low frequency phonons and decreased to a small value above 3–4 THz.

The strong dependence of transmission coefficients on phonon frequency has an important effect on which phonons conduct heat in nanocrystals and hence on strategies to further reduce the thermal conductivity of nanocrystalline silicon. Quantitatively determining which phonons carry heat in nanocrystals requires simulating phonon transport in the structure of a nanocrystalline solid. Several works used a Monte Carlo (MC) algorithm to examine thermal transport in nanostructures including nanocrystals^19^,^20^,^21^,^22^,^23^. However, most of these simulations assumed a grey model for the phonon dispersion due to computational cost excepting the work of Hao *et al*.^19^. Recent advances in variance-reduced Monte Carlo (VRMC) simulations have resulted in significant computational speedups on the order of 10^9^ compared with traditional MC simulation^24^,^25^. These algorithms have been used to study thermal transport in nanomeshes and complex 3D cellular structures^26^,^27^. Hua and Minnich used the method to study phonon transport in Si and SiGe nanocrystals assuming an isotropic phonon dispersion and semi-empirical relaxation times. They reported that low frequency phonons make an unexpectedly large contribution to thermal conductivity in nanocrystals, compared to the prediction of a gray model, due to the high transmission of low frequency phonon modes^23^. However, this work employed a simplified, isotropic dispersion that differs from the actual Si dispersion.

Including the full phonon dispersion with the correct phonon density of states is essential to inferring key properties such as phonon transmission coefficients at the grain boundaries from experimental data^28^,^29^. Several works have reported MC methods that incorporate a full phonon dispersion^30^,^31^, however, these methods are computationally expensive as they do not employ the efficient algorithm of Peraud *et al*.^24^,^25^. Vermeersch *et al*. developed a VRMC method using ab-initio full phonon dispersion to study cross-plane thermal transport in thin films^32^. As yet, no ab-initio based study of phonon transport in nanocrystalline solids needed to identify strategies to further decrease thermal conductivity has been reported.

Here, we examine thermal transport in nanocrystalline Si and SiGe using VRMC simulations based on the full phonon dispersion and intrinsic lifetimes obtained from first-principles. By fitting the transmission coefficient profiles versus phonon frequency, we achieve excellent agreement with experimental thermal conductivity of nanocrystalline Si^17^. The trend of the fitted transmission coefficients is consistent with that obtained experimentally by Hua *et al*.^18^. Based on these calculations, we find that low energy phonons with mean free path longer than grain size transport substantial amounts of heat in nanocrystalline SiGe, even with consideration of electron-phonon scattering, due to their near-unity transmission across grain boundaries. In SiGe with 2% Ge concentration, these phonons contribute over a third of the thermal conductivity. This observation suggests that gains in *ZT* are possible by suppressing the contribution of low energy phonons.

## Method

**Figure 1:**
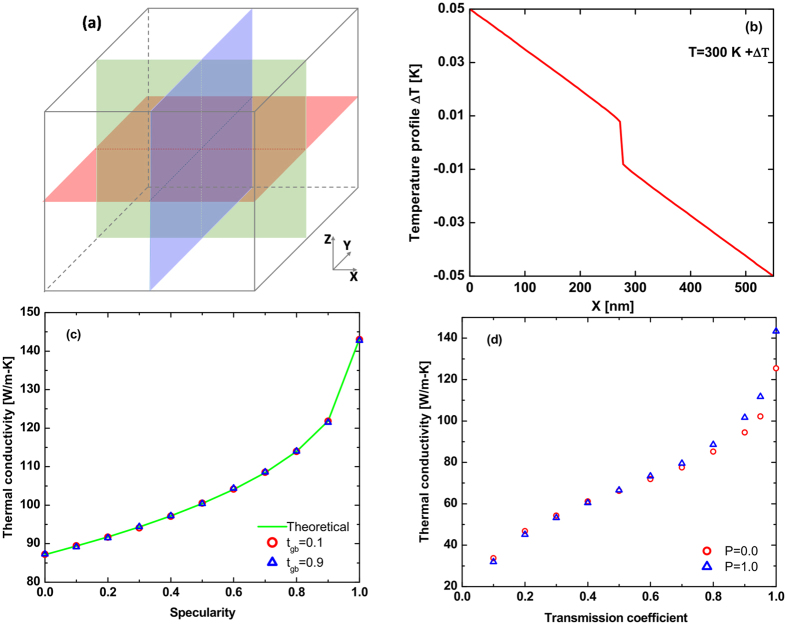
(**a**) Schematic of the 3D cubic simulation box for the nanocrystalline domain. The grain boundaries, which are represented by blue, green and pink planes, are perpendicular to the *x*, *y* and *z* directions, respectively. (**b**) Temperature profile ΔT along *x* direction for nanocrystalline Si with 550 nm grain size at 300 K, which corresponds to the simulation in Fig. 2(c). The temperature difference across the simulation box is 0.1 K. The absolute temperature (*T*) can be obtained by *T* = 300 K + Δ*T*. (**c**) Thermal conductivity versus specularity for only one grain boundary (pink plane) which is parallel to the heat flux (*x* direction) in the simulation box. The two values of constant transmission coefficient considered are 0.1 (red circles) and 0.9 (blue triangles). The parallel grain boundary is only affected by the specularity. (**d**) Thermal conductivity versus transmission coefficient for only one grain boundary (blue plane) which is perpendicular to the heat flux in the simulation box. Two cases of constant specularity, *P* = 0.0 (red circles) and *P* = 1.0 (blue triangles) are studied. The perpendicular grain boundary is mainly affected by the transmission. Constant transmission and specularity is used for all phonon modes in each case. The grain size is 550 nm and the thermal conductivity is calculated at 300 K.

Our geometry for nanocrystalline Si is a 3D cubic simulation box oriented along the coordinates of Brillouin zone as shown in Fig. 1(a). A temperature gradient is applied in the *x* direction to set up a heat flux. We apply a periodic heat flux boundary condition along the *x* direction^19^ and a specular boundary condition in the *y* and *z* directions so that the computational domain represents a unit cell that is repeated in all directions. Three grain boundaries (blue, green and pink plane in Fig. 1(a)) perpendicular to the *x*, *y*, *z* directions bisect the cubic simulation box. Because of symmetry boundary conditions, the size of grain boundaries is the same as that of the cubic simulation domain.

Phonon transport in this domain is described by the Boltzmann transport equation under the relaxation time approximation. The deviational, energy based Boltzmann transport equation is given by (ref. 24)


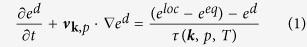


where 
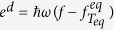
 is the desired deviational distribution function, ***v***_***k**,p*_ is the group velocity, *ω*_***k***_,_*p*_ is the angular frequency, 
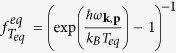
 is the Bose-Einstein distribution at the control temperature *T*_*eq*_, and *τ(**k***, *p*, *T*) is the relaxation time. Here ***k*** and *p* denote the wavevector and polarization of a phonon mode, respectively.

We solve this equation using the linearized version of Peraud and Hadjiconstantinou’s algorithm with a spatially variable equilibrium temperature^25^. In this work, we generalize this algorithm to accept the full phonon dispersion and intrinsic lifetimes for natural bulk Si provided by Lindsay *et al*. We used 16,384 ***k*** points over the entire Brillouin zone, with six polarizations at each ***k***. The number of deviational particles is set as *N*_*ph*_ = 8 · 10^6^ for each simulation. Following the algorithm of ref. 25, these particles are advected through the domain and scattered by internal scattering mechanisms as well as reflected and transmitted by grain boundaries. The net heat flux is obtained by considering the initial and final position of each particle over a defined period of time, from which thermal conductivity is obtained with knowledge of the imposed temperature differential and size of the simulation box. The final reported thermal conductivity is an average of the values obtained from ten simulations. The effective mean free path for each phonon mode in the domain including grain boundary scattering is calculated by averaging the total simulation time over the total number of scattering events that occurred for a phonon mode.

We briefly describe the modifications to the original algorithm^25^ that are necessary to incorporate the full phonon dispersion. In the original algorithm, the isofrequency surface of Brillouin zone is a sphere, and so when a new phonon is drawn after scattering, its traveling direction is chosen isotropically. In the case of a full dispersion with arbitrary anisotropies, this method is no longer appropriate.

The two classes of scattering events we must consider are inelastic scattering, when the phonon frequency is redrawn after internal scattering, or elastic scattering, when the phonon frequency is maintained after grain boundary scattering. To incorporate these scattering event classes, we histogram all of the phonon modes by frequency. As in the original algorithm, if internal scattering occurs we first select the phonon frequency using the inverse transform method^24^. Subsequently, to determine the phonon travelling direction we choose the wave vector ***k*** and polarization *p* by randomly choosing a mode within the selected frequency bin with probability proportional to the weight of the mode *w*_***k***_. In the case of elastic grain boundary scattering, the frequency is maintained and we only perform the latter procedure. Note that for elastic grain boundary scattering, the probability to choose a mode in the frequency bin is weighted by the mode’s component of group velocity parallel to the boundary normal. In the original algorithm, this weight corresponds to a cosine term. The advection and scattering processes in the MC simulation are the same as that in the original algorithm. The only other change is that the volumetric source corresponding to a steady state linear temperature profile follows the distribution, 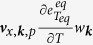 rather than a distribution with a cosine term as the isotropic case.

Phonon transmission and reflection at grain boundaries are the key processes in the nanocrystalline solid that affect thermal conductivity, and there are two parameters that describe these processes. First, the transmission coefficient describes the probability that the phonon transmits through a grain boundary. Second, the specularity parameter describes the probability that the phonon maintains its transverse momentum on scattering. Grain boundary scattering is implemented in the following procedure. Consider that a phonon mode incident on a grain boundary is specified to have a transmission coefficient *t*_*gb*_ and a specularity parameter *P*. When the phonon encounters the grain boundary, a random number is drawn. If the random number is less than the transmission coefficient, the phonon transmits the grain boundary, otherwise the phonon is reflected. Then, a second random number is drawn. If the random number is less than the specularity value, the phonon is specularly transmitted or reflected, otherwise it is diffusely scattered in the forward or backward direction for transmission or reflection, respectively.

The essential inputs to the MC simulation are the transmission coefficients and specularity parameters of phonon modes as a function of their frequencies or wavelengths. In this work, we use spectral profiles of these parameters following the trends of recent experimental measurements. The transmission coefficients are obtained by fitting MC simulation results to the experimental thermal conductivity of nanocrystalline Si^17^. The general trend of transmission coefficients verus frequency follows that of measurements by Hua *et al*.^18^, who found that transmission coefficients between Al and Si interface are near unity for low frequency phonon and decrease rapidly to near zero with increasing frequency^18^. For the specularity parameter, experiments by Ravichandran *et al*. suggest that the specularity at rough silicon boundaries can be specified by Ziman’s specularity, 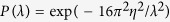, with *η* ~ 0.12 − 0.15 nm^33^. Here 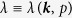 is phonon wavelength and *η* is taken to be an adjustable parameter with a value in the range specified above.

**Figure 2:**
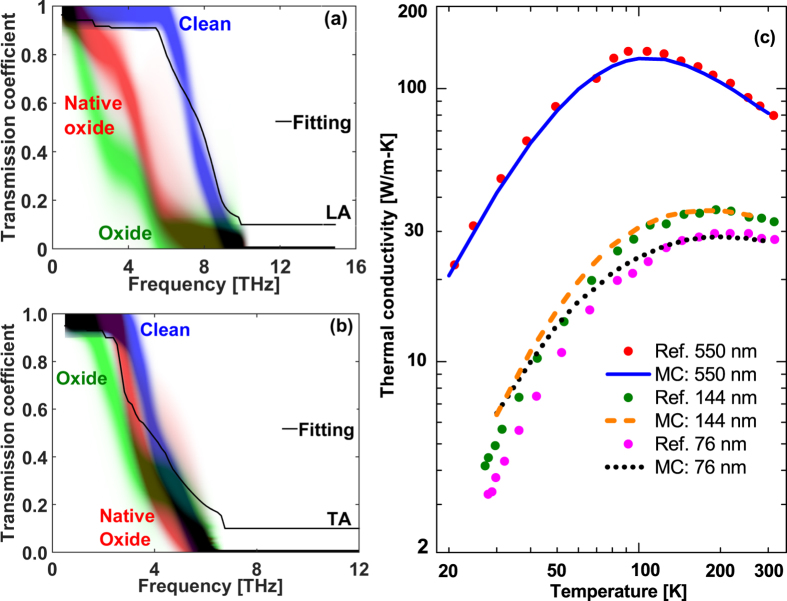
Transmission coefficients versus frequency for (**a**) longitudinal phonons and (**b**) transverse phonons. The solid black line is the transmission coefficients obtained by fitting to experimental data on the 550 nm sample^17^. The blue, red and green shading are representative transmission coefficients from Hua *et al*. for different Al/Si interfaces^18^. Our fitted transmission coefficients are consistent with their results. (**c**) Temperature dependent thermal conductivity for nanocrystalline Si with 550 nm (blue solid line), 144 nm (yellow dashed line) and 76 nm (black dots) grain sizes. The transmission coefficients in (**a**) and (**b**) are applied in all the simulation cases. The parameter *η* for specularity is set to 0.125 nm, 0.26 nm and 0.11 nm to fit the simulation results to the experimental measurements^17^ for 550 nm (red dots), 144 nm (green dots) and 76 nm (pink dots) grain sizes, respectively.

Figure 1 (b) shows the temperature profile ΔT for 550 nm grain size sample at 300 K, which are recorded during the simulation. The absolute temperature (*T*) can be calculated by *T* = 300 K + Δ*T*. The temperature difference across the simulation box is 0.1 K. For this calculation, the domain consisted of the standard cubic simulation box with three grain boundaries, and the transmission coefficients and the specularity are the same as that used in nanocrystalline Si with 550 nm grain size at 300 K in Fig. 2(c).

## Results

We first examine how the different grain boundary orientations affect thermal conductivity by considering domains with a single grain boundary. We took the grain size to be 550 nm and calculated the thermal conductivity for various constant transmission and specularity parameters with only the pink grain boundary, which is parallel to heat flux, or with only the blue grain boundary, which is perpendicular to the heat flux.

Figure 1(c) shows the thermal conductivity of a domain with a parallel grain boundary as a function of specularity calculated using VRMC simulation and a theoretical result based on Fuchs-Sondheimer theory^34^. The thermal conductivity increases with an increase of specularity, however, the thermal conductivity is independent of transmission. Therefore, only the specularity of parallel grain boundaries affects the thermal conductivity. This observation can be understood from symmetry of the grain boundary with respect to the imposed thermal gradient.

Figure 1(d) shows the thermal conductivity of a domain with a perpendicular grain boundary as a function of transmission coefficient. Overall, the specularity has little effect, particularly when the transmission is less than 0.8. The bulk thermal conductivity is achieved when the transmission coefficient and specularity are both equal to unity, as expected. Therefore, for perpendicular grain boundaries, the transmission coefficients are the main factor that modify the thermal conductivity. Comparing the variation in thermal conductivity in Fig. 1(c) and (d) with changes in transmission coefficients and specularity parameters, we found that the perpendicular grain boundaries have a larger effect on the thermal conductivity than the parallel grain boundaries.

With this understanding, we now examine the experimental thermal conductivity measurements of Wang *et al*. on nanocrystalline silicon^17^. In their work, Wang *et al*. observed that the thermal conductivity varies as *T*^2^ at low temperature, indicating the transmission coefficients cannot be a constant for all modes as such a profile would lead to a *T*^3^ trend. Our code, with ab-initio dispersion, allows us to determine which transmission coefficients and specularity parameters are capable of explaining the data.

We fit these data using trends for transmission coefficients and specularity parameters obtained from prior experimental measurement as described in Method section. Figure 2(a) and (b) shows the fitted transmission coefficients for the 550 nm grain size sample versus frequency for longitudinal and transverse acoustic phonons. The data of Hua *et al*.^18^ are plotted for comparison and agree reasonably well with the fitted profile. The resulting thermal conductivity versus temperature for the 550 nm grain size sample is given in Fig. 2(c). The fitted specularity parameter yields *η* = 0.125 nm, which is consistent with the measurement by Ravichandran *et al*.^33^. The self-consistency of these results provide evidence that the transmission coefficient and specularity parameters obtained in refs 18 and 33 are correctly describing the phonon interactions with grain boundaries.

Using the same transmission coefficients we fit the specularity of the nanocrystalline Si with grain sizes 144 nm and 76 nm at 300 K, yielding *η* = 0.26 nm and *η* = 0.11 nm for grain sizes 144 nm and 76 nm, respectively. As shown in Fig. 2(c), the thermal conductivities of the MC simulation are consistent with the measurements when the temperature is larger than 100 K. The thermal conductivity of MC simulation shows some discrepancy with the measurements as the temperature decreases but the trend is not dissimilar from the experiment. To facilitate subsequent comparisons between different grain sizes, we use the same transmission coefficients and specularity obtained from the 550 nm sample for all further calculations.

The fitted transmission coefficients and specularity parameters thus indicate that low frequency phonons experience high transmission at frequencies less than 2–3 THz while high frequency phonons above around 4 THz are largely reflected. We now examine the impact of this strong frequency dependence on the phonon modes responsible for heat conduction in the nanocrystals.

**Figure 3:**
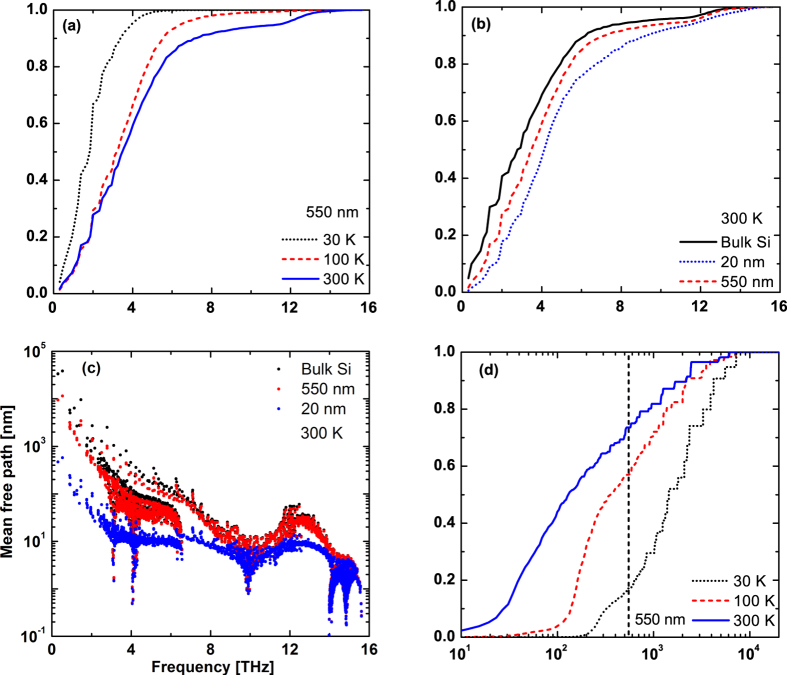
(**a**) Normalized accumulation of thermal conductivity of 550 nm grain size versus frequency for 30 K (black dotted line), 100 K (red dashed line), 300 K (blue line). (**b**) Normalized accumulation of thermal conductivity versus frequency for bulk Si (black line), nanocrystalline Si with 550 nm (red dashed line), 20 nm (blue dotted line) grain sizes at 300 K using the fitted transmission and specularity for the 550 nm sample. The thermal conductivity is mainly contributed from low frequency phonons. (**c**) Mean free paths of each phonon mode for bulk Si (black dots), nanocrystalline Si with 550 nm (red dots), 20 nm (blue dots) grain sizes at 300 K. (**d**) Normalized accumulation of thermal conductivity versus mean free path for nanocrystalline Si with 550 nm grain size at 30 K (black dotted line), 100 K (red dashed line), 300 K (blue line). Low frequency phonons with mean free path exceeding the grain size in nanocrystalline Si have a major contribution to the thermal conductivity.

We present the thermal conductivity as a function of frequency in Fig. 3(a) and (b). Figure 3(a) compares the accumulation of thermal conductivity of nanocrystalline Si with the 550 nm grain size at 30 K, 100 K, and 300 K. At 300 K, phonons with frequency less than 4 THz contribute 60% in nanocrystalline Si with 550 nm grain size, nearly the same amount as in bulk Si, 69%. In addition, the contribution of low frequency phonons increases as the temperature decreases in nanocrystalline Si. Figure 3(b) shows that similar trends are observed for the 20 nm grain size, while the contribution from low frequency phonons is decreased in nanocrystalline Si with smaller grain sizes. The thermal conductivity of nanocrystalline Si with 20 nm grain size decreases to 8.4 W/m-K.

Figure 3(c) shows the mean free path for each phonon mode versus frequency. The longest mean free path in nanocrystalline Si with 550 nm grain size is around 33% of that in bulk Si, approximately 11 microns, but is still much larger than the grain size. The mean free path of high frequency phonons is not strongly affected by grain boundary scattering, as expected, because of the high phonon-phonon scattering rate for high frequency phonons. The normalized accumulation of thermal conductivity versus mean free path, Fig. 3(d), demonstrates that the contribution of phonons with mean free path larger than grain size is 26.7% for 550 nm, respectively, at 300 K. In addition, the contribution from these phonons increases as the temperature decreases.

It is interesting to note the outsize role of low energy phonons to thermal conductivity. In bulk Si at 300 K, the fraction of the number of phonon modes with mean free path larger than 550 nm is 0.73% yet they contribute almost 50% to the thermal conductivity. This outsize role remains in nanocrystalline Si: we find that the fraction of the number of phonon modes with larger mean free path than the grain size is 0.38% and 1.92% for 550 nm and 20 nm grain sizes, respectively, yet these modes contribute nearly a third to the total thermal conductivity. Therefore, very small portion of phonons is responsible for a major fraction of the thermal conductivity, suggesting that disrupting these modes will lead to large reductions in thermal conductivity.

**Figure 4:**
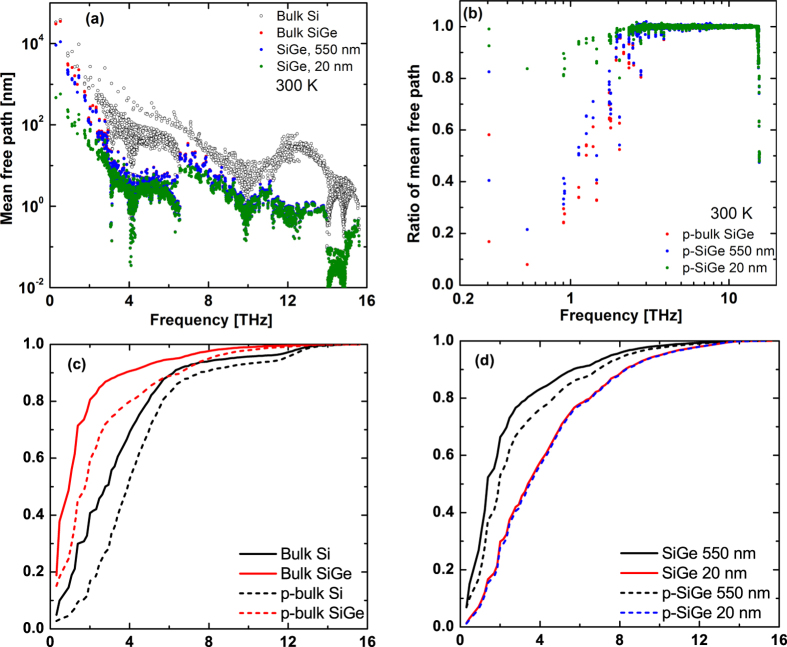
(**a**) Mean free path of each phonon mode versus frequency for undoped bulk SiGe (red dots), nanocrystalline SiGe with 550 nm (blue dots) and 20 nm (green dots) grain size at 300 K. (**b**) Ratio of mean free path for each phonon versus frequency for p-type doped bulk SiGe (red dots), nanocrystalline SiGe with 550 nm (blue dots) and 20 nm (green dots) grain sizes, to the corresponding mean free path of the undoped materials in (**a**). (**c**) Accumulation of thermal conductivity of undoped bulk Si (black line), undoped bulk SiGe (red line), p-type bulk Si (black dashed line) and p-type bulk SiGe (red dashed line) versus frequency. (**d**) Accumulation of thermal conductivity of undoped nanocrystalline SiGe with 550 nm (black line), 20 nm (red line) grain sizes, and p-type nanocrystalline Si with 550 nm (black dashed line) and 20 nm (blue dashed line) versus frequency. The mean free paths of high frequency phonons are decreased by Ge defect scatterings, and the mean free paths of very low frequency phonons are decreased by the electron-phonon scattering.

We now use our simulations to examine phonon transport in nanocrystalline SiGe thermoelectrics. These materials have two additional scattering mechanisms, point defect scattering and electron-phonon scattering, compared to nanocrystalline Si. We first account for mass defect scattering rate using the Tamura formula^35^ and add it to the intrinsic phonon-phonon scattering rate by Matthiessen’s rule neglecting the coupling between phonon-phonon scatterings and defect scatterings^36^. For SiGe with 2% Ge concentration, we obtain a bulk thermal conductivity of around 34.3 W/m-K at 300 K, a value that is consistent with previous work^37^. Taking the transmission coefficients and specularity parameters to be the same as those of nanocrystalline Si with 550 nm grain size, we find that point defect scattering results in a decrease in thermal conductivity from 34.3 (bulk SiGe) to 18.8 and 4.6 W/m-K for 550 nm and 20 nm grain sizes, respectively. The origin of this decrease can be identified from Fig. 4(a), which shows that point defect scattering strongly scatters high frequency phonons but has only minimal effect of phonons with frequency less than 2 THz. In nanocrystalline SiGe, the longest mean free path is approximately 11 microns and 573 nm for 550 nm and 20 nm grain sizes, respectively. Thus, the longest mean free path is decreased almost one order compared with that in bulk SiGe but is again much larger than the grain sizes.

Second, electron-phonon interactions can significantly decrease the thermal conductivity of Si due largely to scattering of low frequency phonons^38^. We obtained ab-initio electron-phonon scattering rates for both p-type and n-type doping with a concentration of 10^20^ cm^−3^ from Liao *et al*.^38^. This scattering mechanism results in an additional reduction of thermal conductivity of p-type nanocrystalline SiGe. The decrease is more severe for the 550 nm grain size, from 18.8 to 13.2 W/m-K, than for the 20 nm grain size, from 4.6 to 4.4 W/m-K. This observation can be explained from Fig. 4(b), which compares the mean free path ratio, relative to those of the corresponding undoped materials, of each phonon for p-type bulk SiGe and p-type nanocrystalline SiGe with 550 nm and 20 nm grain sizes. The figure shows that low frequency phonons are strongly scattered in the 550 nm sample while they are minimally affected in the 20 nm sample, indicating that the electron-phonon scattering rate is small compared to the grain boundary scattering rate in the latter case. Therefore, the relative effect of electron-phonon interactions is smaller in nanocrystalline solids with smaller grains. Similar trends are observed for n-type doping.

The effect of these scattering mechanisms on thermal conductivity is presented in Fig. 4(c) and (d), which shows the normalized accumulation of thermal conductivity of undoped and p-type bulk and nanocrystalline Si and SiGe versus frequency. As the high frequency phonons are strongly scattered by Ge point defects, the relative contribution of low frequency phonons in bulk SiGe to the thermal conductivity increases compared to that of bulk Si. Due to the electron-phonon scattering for low frequency phonons, p-type bulk Si and SiGe has a smaller contribution from low frequency phonons compared with their undoped counterparts. A similar trend is shown in Fig. 4(d) for nanocrystalline Si with 550 nm grain size. The contribution from low frequency phonon remains almost unchanged in p-type nanocrystalline SiGe with 20 nm grain size compared with that of undoped material, indicating that grain boundary scattering dominates in smaller grain size nanocrystalline SiGe.

**Figure 5:**
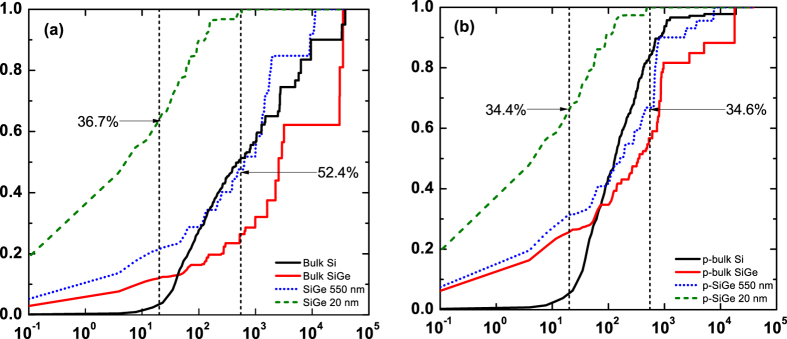
Normalized accumulation of thermal conductivity of (**a**) undoped and (**b**) p-type bulk Si (black line), SiGe (red line) and nanocrystalline SiGe with 550 nm (blue dotted line) and 20 nm (green dashed line) grain size at 300 K. The vertical dashed lines in (**a**) and (**b**) represent 550 nm and 20 nm grain sizes, respectively. Phonons with mean free path larger than grain size contribute 36.7% and 52.4% for undoped SiGe with 20 nm and 550 nm grain size, respectively, and the contribution is 34.4% and 34.6% for doped SiGe. A small portion of phonons with long mean free paths still contributes substantially to the thermal conductivity of p-type SiGe, indicating potential for improving *ZT* by reducing lattice thermal conductivity.

Finally, we show the normalized thermal conductivity accumulation for undoped and p-type materials in Fig. 5(a) and (b), respectively. Compared with bulk Si and nanocrystalline SiGe, bulk SiGe has the largest contribution from phonons with large mean free path. Even in the doped samples with electron-phonon interactions, the fraction of the number of phonon modes with mean free path larger than the grain size is 0.11% and 0.82% for 550 nm and 20 nm grain sizes, respectively, yet in both cases these low energy modes contribute nearly a third of the total thermal conductivity. We reach a similar conclusion for n-type doping. Therefore, our ab-initio based analysis shows that potential for improving *ZT* remains by reducing lattice thermal conductivity in nanocrystalline SiGe alloys.

## Conclusion

We studied thermal transport in nanocrystalline Si with an ab-initio based Monte Carlo method with the full phonon dispersion and intrinsic lifetimes obtained from first-principles. We find that the high transmission of low frequency phonons across the grain boundary leads to a small fraction of the modes contributing substantially to the thermal conductivity of nanocrystalline Si. This conclusion remains true even in nanocrystalline SiGe despite point-defect and ab-initio electron-phonon scattering, indicating that the key to further reducing lattice thermal conductivity is to disrupt the transport of low energy phonons. Our study also shows the powerful insights that can be obtained with ab-initio based phonon transport modeling of complex nanostructures with a minimum of adjustable parameters.

## Additional Information

**How to cite this article**: Yang, L. and Minnich, A. J. Thermal transport in nanocrystalline Si and SiGe by *ab initio* based Monte Carlo simulation. *Sci. Rep.* **7**, 44254; doi: 10.1038/srep44254 (2017).

**Publisher's note:** Springer Nature remains neutral with regard to jurisdictional claims in published maps and institutional affiliations.
