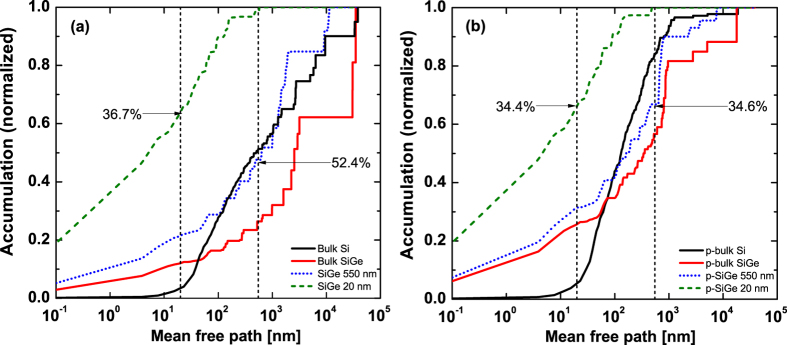# Corrigendum: Thermal transport in nanocrystalline Si and SiGe by *ab initio* based Monte Carlo simulation

**DOI:** 10.1038/srep46771

**Published:** 2017-05-02

**Authors:** Lina Yang, Austin J. Minnich

Scientific Reports 7: Article number: 4425410.1038/srep44254; published online: 03 14 2017; updated: 05 02 2017

This Article contains errors in Figures 3, 4 and 5 where the graphs were labelled incorrectly. The correct Figures 3, 4 and 5 appear below as Figures 1, 2 and 3 respectively.

## Figures and Tables

**Figure 1:**
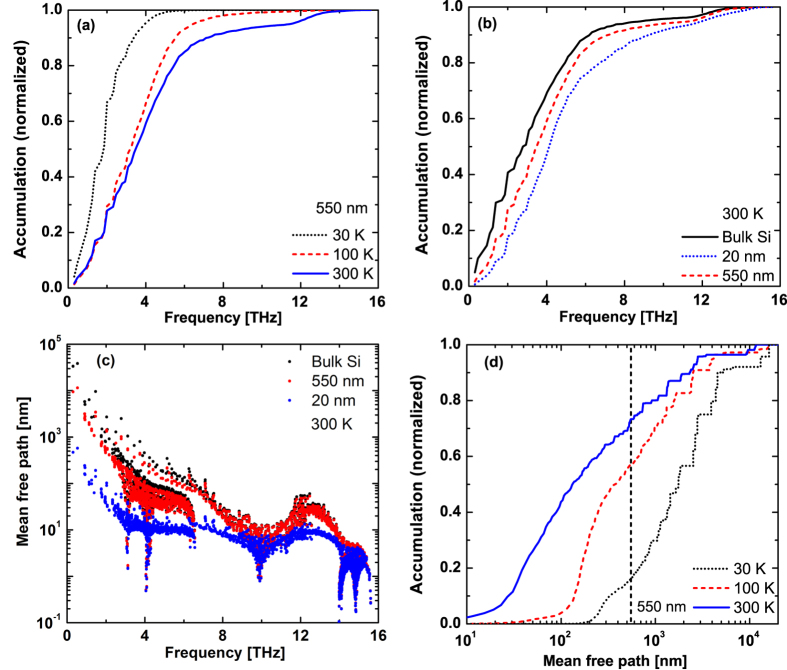


**Figure 2:**
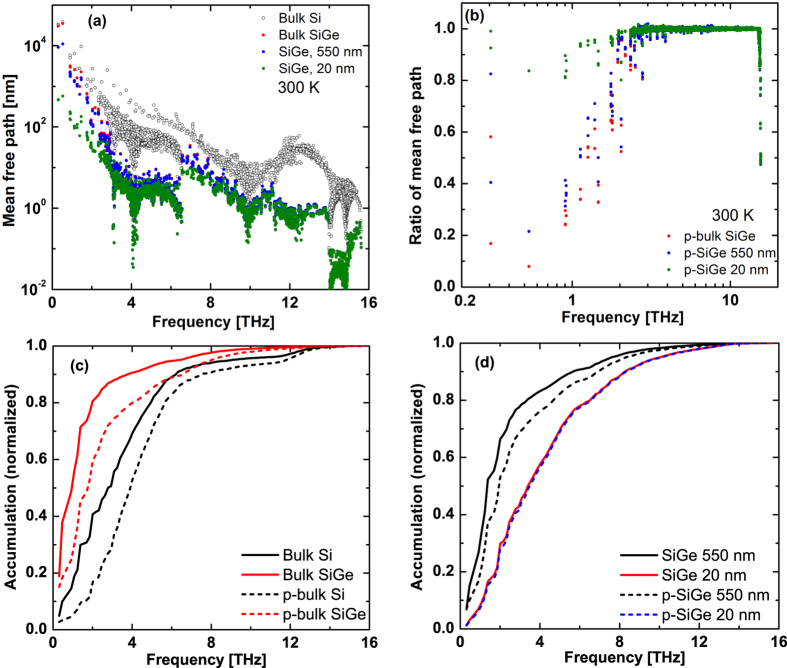


**Figure 3:**